# Cognitive Tele-Enhancement in Healthy Older Adults and Subjects With Subjective Memory Complaints: A Review

**DOI:** 10.3389/fneur.2021.650553

**Published:** 2021-07-05

**Authors:** Cristina Alaimo, Elena Campana, Maria Rachele Stoppelli, Elena Gobbi, Francesca Baglio, Federica Rossetto, Giuliano Binetti, Orazio Zanetti, Rosa Manenti, Maria Cotelli

**Affiliations:** ^1^Neuropsychology Unit, IRCCS Istituto Centro San Giovanni di Dio Fatebenefratelli, Brescia, Italy; ^2^IRCCS, Fondazione don Carlo Gnocchi—ONLUS, Milan, Italy; ^3^Macroattività Ambulatoriale Complessa (MAC) Memory Clinic and Molecular Markers Laboratory, IRCCS Istituto Centro San Giovanni di Dio Fatebenefratelli, Brescia, Italy; ^4^Alzheimer's Research Unit, Macroattività Ambulatoriale Complessa (MAC) Memory Clinic, IRCCS Istituto Centro San Giovanni di Dio Fatebenefratelli, Brescia, Italy

**Keywords:** cognitive, telerehabilitation, tele-enhancement, healthy older adults, subjective memory complaints

## Abstract

**Background:** In recent years, emphasis has been placed on cognitive enhancement to stimulate cognitive abilities and prevent functional decline. Considering that traditional face-to-face interventions can be very expensive and are not accessible to all individuals, the need to transfer care from the clinic to the patient's home is evident. In this regard, cognitive tele-enhancement interventions have received increased attention.

**Aim:** The aim of this review was to provide an overview of protocols that apply remotely controlled cognitive training with individualized feedback on performance by the therapist in healthy older adults or participants with subjective memory complaints.

**Methods:** Out of 35 articles assessed for eligibility, eight studies were identified. Of the selected studies, five included cognitively healthy older adults, while three included participants with subjective memory complaints.

**Results:** Most of the reviewed studies showed beneficial effects of cognitive tele-enhancement interventions, reporting improvements in memory, sustained attention, working memory, executive functions, and language abilities. Moreover, reductions in anxiety and depression symptomatology levels, as well as in subjective memory difficulties, were described in some of the studies.

**Conclusions:** Cognitive tele-enhancement treatment could be a good alternative to face-to-face intervention. This literature review highlights the importance of applying preventive cognitive interventions to subjects with initial subjective memory complaints. Remote modalities seem to facilitate the application of such interventions.

## Introduction

The world's population is aging, requiring us to face new challenges. Improvements in medical care have led to a consistent increase in the average age, and it is estimated that the elderly population will increase in the future (1, 2). In Europe, the proportion of people aged 65 or over is expected to double in the next 25 years (3).

Healthy cognitive functioning is a primary condition of well-being, independence, and successful aging (4–6). In recent years, promoting active and healthy aging has become an important challenge to ensure good physical, social, and cognitive functioning. However, aging is often associated with cognitive decline related to impairment of memory, language, judgment, and executive functions (7). In light of these considerations, it is obvious that the most at-risk population is elderly individuals, who often report memory complaints in the absence of objective deficits from neuropsychological assessment and psychometric tests (8). Although these cases have been extensively described, it is only in recent years that this condition, better known as “subjective memory complaints” (SMCs), has received increasing interest as a consequence of greater attention in characterizing the preclinical stages of Alzheimer's disease (AD) (9). Indeed, many epidemiological studies have shown that this category of patients is associated with a higher risk of developing mild cognitive impairment (MCI) or AD (10).

At present, pharmacological therapies to prevent cognitive decline have not had great success, and, consequently, much emphasis has been placed on cognitive training to stimulate cognitive abilities and prevent functional decline. The term “brain plasticity” is used to refer to the ability of the human brain to develop continuously through daily experience and the active maintenance of cognitive functions (11–13) and, from a neurorehabilitative perspective, to respond to intrinsic or extrinsic stimuli by reorganizing its structure, function, and connections (14). Several studies have indicated a wide range of activities that can induce brain plasticity; among these, physical exercise and cognitive stimulation were the most suitable (15, 16). Cognitive training refers to a standardized and systematic set of exercises aimed at stimulating specific cognitive domains: attention, memory, language, and executive functions (17, 18). Cognitive training is a promising approach for slowing cognitive decline and provides the potential to generalize the benefits to other domains that are not directly stimulated and to maintain the results over time (19–24). Traditional forms of cognitive programs are delivered in person by a therapist, but traditional face-to-face treatment can be very expensive and is not easily accessible due to several barriers, such as difficulty in reaching the therapist's office, physical or financial difficulties, or lack of caregivers (25). In the last few years, the need to transfer care from the clinic to the patient's home to overcome these barriers and to introduce sustainable and accessible care treatments for all has become increasingly evident (26–28). The World Health Organization defines “telemedicine” as “*the delivery of health care services, where distance is a critical factor, by all health care professionals using information and communication technologies for the exchange of valid information for diagnosis, treatment and prevention of disease and injuries, research and evaluation, and for the continuing education of health care providers, all in the interest of advancing the health of individuals and their communities*” (29).

A subcategory of the broader concept of telemedicine is telerehabilitation. The term telerehabilitation commonly refers to any type of home-based intervention in which digital technology (mobile phones, video, sensors, online platforms, etc.) allows for a double communication loop between the hospital and the patient (30). The presence of the “double loop” represents an essential requirement of telerehabilitation that enables both remote monitoring of patient performance and responding with appropriate feedback (26, 30). Recently, with the advancement of technology and following the elimination of constraints due to temporal and spatial limits, “cognitive telerehabilitation” has become more popular in the fields of cognitive assessment and/or intervention for patients with neurological disorders (26, 27, 31, 32). Dodakian et al. (33) tested an asynchronous home-based telerehabilitation program in patients with stroke and found good compliance by participants; likewise, a randomized controlled trial involving subjects with MCI or mild-to-moderate AD used a tablet to deliver an asynchronous, personalized rehabilitation plan with both cognitive and aerobic physical exercise providing feedback through weekly phone calls with the aim of observing the efficacy of a technology-enhanced home care service to preserve cognitive and motor levels of functioning (34). Most importantly, as Cotelli et al. (31) showed in their systematic review, telerehabilitation can be as effective as face-to-face interventions. Many studies have shown that a key component of the successful application of cognitive telerehabilitation systems is the use of feedback from the therapist on the performance ability of patients, which has a positive influence on their performance, engagement, and motivation (35). In fact, clinicians, even if at distance, using synchronous (in which patient and clinician perform activities in real-time, for example, through videoconference) or asynchronous (in which patient and therapist work offline with virtual therapists or digital contents) treatment modalities, can directly work with patients to set goals that are reasonable and achievable. The possibility of digital contents, both to collect several parameters associated with user's performance and to implement adaptive training, allows reaching a balance between individual resources and the difficulty level of rehabilitative activities also in the asynchronous telerehabilitation model. Moreover, the asynchronous modality permits the patient to organize their time to dedicate some to rehabilitation with a certain degree of freedom, allowing the therapist to prescribe high-intensity training for the long-term in a wide number of older adults (34, 36). In this way clinicians can help subjects believe they have the skills and ability to reach their goals and, finally, provide positive reinforcement (37) using online or offline monitoring and feedback modalities.

Although this training was initially mainly dedicated to neurological patients, the focus has shifted to the prevention of cognitive decline (38, 39). Accordingly, a less investigated but certainly no less interesting area is the application of tele-enhancement cognitive interventions following the positive trajectory of aging in older adults who are not necessarily subject to cognitive and/or physical decline. In light of new technologies, cognitive training can now be administered remotely as a nonpharmacological treatment in patients with dementia (31) and as a preventative intervention in healthy adults. Thus, the aim of this review was to identify and provide an overview of treatments in which the double loop communication approach was implemented (i.e., cognitive training remotely controlled by a therapist and in which feedback on training performance was given) in healthy older adults and in participants with SMC.

## Review of the Literature

### Search Strategies and Study Selection Criteria

**Figure 1:**
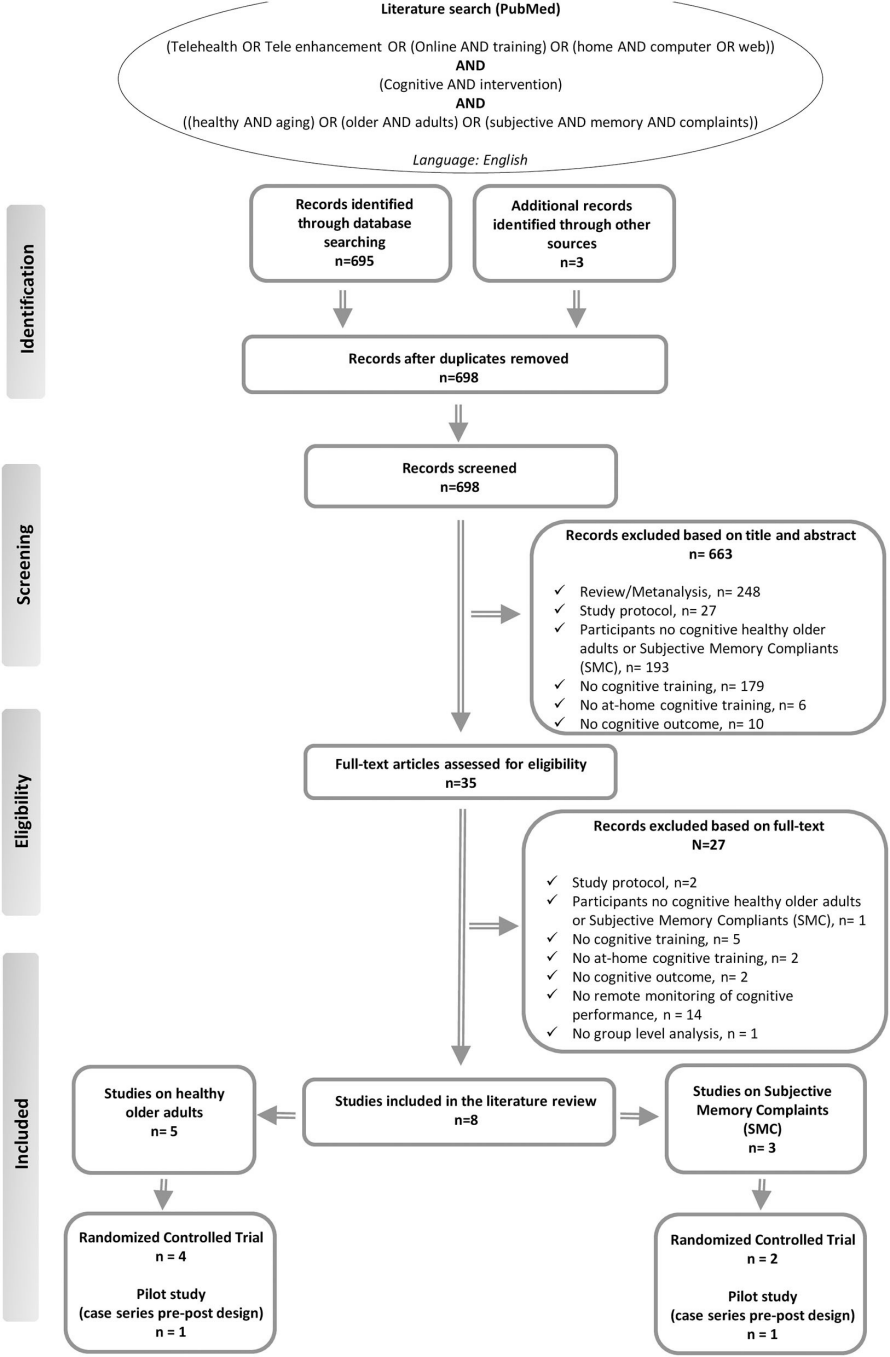
Summary of the literature search—PRISMA flow diagram (48).

We searched the literature in the Medline (PubMed) database using the following terms: “[*Telehealth OR Tele enhancement OR (Online AND training) OR (home AND computer OR web)*] *AND (Cognitive AND intervention) AND* [*(healthy AND aging) OR (older AND adults) OR (subjective AND memory AND complaints)*].” We reviewed all titles and abstracts and examined all relevant original research articles. Additionally, the references of the articles were examined to identify possible additional sources. Only English-language articles were selected (see flowchart in Figure 1). For study selection, we primarily analyzed the title and abstract of the paper. From this first analysis, animal studies, reports of secondary data, such as meta-analyses, reviews, or letters, usability studies, and study protocols were excluded. Thereafter, we proceeded to read the full texts of the remaining articles. The following criteria were applied in the final selection of the studies: (a) original research; (b) conducted on cognitively healthy older adult subjects and/or participants with SMC; (c) comprising at-home tele-enhancement cognitive interventions; (d) providing at least one cognitive outcome; (e) comprising a group level analysis; and (f) published before September 29, 2020. Moreover, studies that included only participants with objective cognitive decline or subjects with other neurological or psychiatric disorders, as well as studies in which cognitive training was not administrated, were not selected for our review. In addition, we excluded studies that did not investigate cognitive training gains through quantitative or subjective cognitive measures. Based on the concept of tele-enhancement, moreover, we also excluded studies that did not provide the possibility for the research assistant to remotely monitor the training and give personalized feedback on training performance to the participant (see in Figure 1).

### Baseline Characteristics of Included Studies

Out of 35 full-text articles assessed for eligibility, only eight studies published between 2012 and 2019 fulfilled the criteria for inclusion in this literature review. Of the selected studies, five included cognitively healthy older adults, while three included participants with SMC. In all selected studies, at least one group in which the participants' training performances were remotely monitored by the therapist was present.

Summarizing, a total of 461 cognitively healthy older adults were enrolled in five selected studies (see Table 1).

**Table 1:** Studies that have assessed the effects of cognitive tele-enhancement interventions on cognitive functions in healthy older adults.

**Study**	**Number and type of subjects (Age: Mean age in years ($\bar{x}$) and SD; Education: Mean years of education ($\bar{x}$) and SD)**	**Study design**	**Intervention system: modality and description (numbers/durations of sessions)**	**Remote control modality for tele-enhancement intervention (monitoring and feedback)**	**Follow-up (yes/none, months)**	**Results**
Brehmer et al. (20)	100 healthy adults (younger and older): - 55 younger healthy adults (**age**: $\bar{x}$ = 26.0; **education:** $\bar{x}$ = 15.0) - 45 older healthy adults (**age:** $\bar{x}$ = 63.8; **education:** $\bar{x}$ = 15.4)	Randomized controlled trial	Single-user, asynchronous, at-home computerized adaptive WM training with Cogmed QM **Duration:** Twenty to twenty-five sessions (26 min, 5 weeks) vs. Single-user, asynchronous at-home computerized nonadaptive low-level WM practice with Cogmed QM **Duration:** Twenty to twenty-five sessions (26 min, 5 weeks)	Monitoring: offlineFeedback: offline	Yes, 3	Both younger and older adults improved their training performance across the sessions of adaptive WM training. Results on **criterion tasks**: Both younger and older adults gained more after adaptive than low-level practice WM training in: - Span Board forward; - Digit Span backward; Results on **near-transfer tasks**: Both younger and older adults gained more after adaptive than low-level practice WM training in: - Span Board backward; - Digit Span forward; Results on **far-transfer tasks**: Both younger and older adults gained more after adaptive than low-level practice WM training in: - Paced Auditory Serial Addition Task (PASAT); - Cognitive Failures Questionnaire (CFQ); Similar performance improvements for the adaptive and the low-level practice training in: - Stroop-color word task; - Raven's Progressive Matrices; Overall, in all tasks, younger participants showed a larger improvement than older adults. Gains maintained at **follow-up** in: - Span Board forward; - Digit Span backward; - Span Board backward; - Digit Span forward; - Stroop-color word task; - Raven's Progressive Matrices; - PASAT; - CFQ.
Anguera et al. (43)	**Experiment 2:** 46 healthy older adults (**age:** $\bar{x}$ = 67.1, SD = 4.2; **education:** $\bar{x}$ = 16.8): - 16 in multitasking training (MTT) group - 15 in active control singletask training (STT) group - 15 in no-contact control (NCC) group	Randomized controlled trial	**MTT and STT groups:** Single-user, asynchronous, at-home computerized adaptive cognitive control training with NeuroRacer **Duration:** Twelve sessions (60 min, 4 weeks) vs. **NCC group:** No training	Monitoring: offline Feedback: offline	Yes, 6	**Both MTT and STT groups** improved in: - Training performance; Only the **MTT group** improved in: - Delayed-recognition working memory tasks; - Test of Variables of Attention (T.O.V.A); Gains maintained at **follow-up:** - Training performance only for MTT group.
Vermeij et al. (40)	23 healthy older adults (**age:** $\bar{x}$ = 70.1, SD = 5.4; **education:** $\bar{x}$ = 14.4, SD = 3.2)	Pilot Study (case series pre-post design)	Single-user, asynchronous, at-home computerized adaptive WM training with Cogmed QM	Monitoring: offline Feedback: offline	Yes, 3	Healthy older adults: Improvement in training performance across the sessions of adaptive WM training (larger than MCI subjects).
	18 MCI (**age:** $\bar{x}$ = 68.4, SD = 6.3; **education:** $\bar{x}$ = 13.6, SD = 3.1)		**Duration:** Twenty-five sessions (45 min, 5 weeks)			**Results on near-transfer tasks**, improvement in: - WAIS-III Digit Span forward; - WMS-III Spatial Span backward; **Results on far-transfer tasks**, improvement in: - Ruff Figural Fluency Test (RFFT); - Personalized goals (as subjective measure of training gains) Gains maintained at **follow-up** in: - WAIS-III Digit Span forward; - WMS-III Spatial Span backward; - RFFT; - Personalized goals (as subjective measure of training gains); MCI subjects Improvement in training performance across the sessions of adaptive WM training. **Results on-transfer tasks**, improvement in: - WAIS-III Digit Span backward; - WMS-III Spatial Span backward; **Results on far-transfer tasks**, improvement in: - RFFT; - Personalized goals (as subjective measure of training gains); Gains maintained at **follow-up** in: - WAIS-III Digit Span backward; - WMS-III Spatial Span backward; - Personalized goals (as subjective measure of training gains);
Buitenweg et al. (21)	139 healthy older adults (**age:** $\bar{x}$ = 67.7; **education:** $\bar{x}$ = 5.8): - 56 in frequent switching (FS) group - 33 in infrequent switching (IS) training group - 50 in mock training (MT) active control group	Randomized controlled double—blind design	**FS and IS groups:** Single-user, asynchronous at-home computerized adaptive multi-domain training with www.braingymmer.com **Duration**: Sixty sessions (30 min, 12 weeks) vs. **MT group:** Single-user, asynchronous at-home computerized nonadaptive executive functions training with www.braingymmer.com **Duration:** Sixty sessions (30 min, 12 weeks)	Monitoring: offline Feedback: offline	Yes, 1	Improvement in training performance across the sessions in all groups (FS and IS groups improved significantly more than MT group); **All three groups** improved in: - Switch task; - Dual task; - Delis—Kaplan Executive Function System (D-KEFS) Trail Making Test-4 (TMT-4); - TMT-B—online version; - Drag- and-drop; - Drag-to-grid; - Click task; - TMT-A- online version; - Digit Symbol Coding (DSC); - DSC—online version; - Tower of London (ToL); - Shipley Institute of Living Scale (SILS); - PASAT; - Rey Auditory Verbal Learning Test (RAVLT); Gains maintained at **follow-up** in: - Switch task; - TMT-B; - Drag- and-drop; - Drag-to-grid; - Click task; - TMT-A; - DSC—online version; - ToL.
Rebok et al. (18)	208 healthy older adults (**age:** $\bar{x}$ = 71.1, SD = 5.0; **education:** $\bar{x}$ = 15.5, SD = 3.2): - 70 in web-based training group - 69 in classroom training group - 69 in wait-list control group	Randomized controlled trial	Single-user, asynchronous at-home web-based mnemonic skills training **Duration:** Ten sessions (60–75 min, 5–6 weeks) vs. Synchronous classroom mnemonic skills training **Duration:** Ten sessions (60–75 min, 5–6 weeks) vs. No training	Monitoring: online Feedback: a facilitator was available (by phone or Internet)	Yes, 6	**All three groups** improved in: - RAVLT; - Rivermead Behavioral Memory Test (RBMT) - Paragraph Recall; - Fluency test; - Animal naming; - Word series; - Digit Symbol Substitution test (DSST); - Metamemory in Adulthood (MIA); - Memory Functioning Questionnaire (MFQ); Gains maintained at **follow-up** in: - RAVLT; - RBMT—Paragraph Recall; - Fluency test; - Animal naming; - Word series; - DSST - MIA; - MFQ; Web-based training group and classroom training group reported satisfaction with the training program

*$\bar{x}$, Mean; CFQ, Cognitive Failures Questionnaire; D-KEFS, Delis-Kaplan Executive Function System; DSC, Digit Symbol Coding; DSST, Digit Symbol Substitution test; FS, frequent switching; IS, infrequent switching; NCC, No-Contact Control; MCI, Mild Cognitive Impairment; MFQ, Memory Functioning Questionnaire; MIA, Metamemory in Adulthood; min, minutes; MT, mock training; MTT, multitasking training; PASAT, Paced Auditory Serial Addition Task; RAVLT, Rey Auditory Verbal Learning Test; RBMT, Rivermead Behavioral Memory Test; RFFT, Ruff Figural Fluency Test; SD, Standard Deviation; SILS, Shipley Institute of Living Scale; STT, Singletask training; TMT, Trail Making Test; ToL, Tower of London; T.O.V.A, Test Of Variables of Attention; WM, Working Memory; WMS-III, Wechsler Memory Scale—Third Edition; WAIS-III, Wechsler Adult Intelligence Scale—Third Edition*.

Four out of five studies on cognitively healthy older adults are Randomized Controlled Trial (RCT) and have an active or passive control group; whereas one is a pilot study (case series pre-post design) without a control group (40). In all these studies, the effects of a single-user and asynchronous tele-enhancement approach based on computerized cognitive training were investigated. Moreover, the tele-enhancement interventions were remotely offline controlled in all studies. Regarding the feedback, it was provided offline in most of the studies included, except for Rebok et al. (18), where a facilitator was available by phone or Internet for assistance during the training. The effects of the computerized cognitive treatment were evaluated on neuropsychological test; moreover, subjective measures were included in the studies of Rebok et al. (18), Brehmer et al. (20), and Vermeij et al. (40). Furthermore, all the selected works investigated the maintenance of the training gains over time (from 1 to 6 months follow-up).

Regarding the studies on SMC participants, a total of 175 subjects were enrolled in the three selected articles (see Table 2). Two randomized controlled trials with at least a control group (active or passive) (38, 41) and one pilot study (case series pre-post design) without a control group were included (42). All the selected studies investigated the effectiveness of a tele-enhancement approach based only on computerized cognitive training, except for the study of Pereira-Morales et al. (41), in which one experimental group (IPP, Integrated Psycho-stimulation Program group) received computerized cognitive training associated with traditional pen and paper cognitive training. In addition, in all studies, the modality for tele-enhancement intervention was single-user and asynchronous with remote offline monitoring and feedback. Furthermore, all studies examined the training effects on cognitive objective and subjective measures. Finally, none of the included studies investigated the long-term effects of the treatment.

**Table 2:** Studies that assessed the effects of tele-enhancement cognitive interventions on cognitive functions in subjects with subjective memory complaints.

**Study**	**Number and type of subjects (Age: Mean age in years ($\bar{x}$) and SD; Education: Mean years of education ($\bar{x}$) and SD)**	**Study design**	**Intervention system: modality and description (numbers/durations of sessions)**	**Remote control modality for tele-enhancement intervention (monitoring and feedback)**	**Follow-up (yes/none, months)**	**Results**
Oh et al. (38)	53 older adults with SMC (**age:** $\bar{x}$ = 59.3; **education:** $\bar{x}$ = 13.9): −18 in Smartphone-based brain Anti-aging and memory Reinforcement Training (SMART) group - 19 in Fit Brains® training group - 16 in wait-list control group	Randomized controlled trial	Single-user, asynchronous, at-home smartphone adaptive (SMART) multi-domain training **Duration:** Forty sessions (15–20 min, 8 weeks) vs. Single-user, asynchronous, at-home adaptive smartphone (Fit Brains®, www.fitbrains.com) multi-domain training **Duration:** Forty sessions (15–20 min, 8 weeks) vs. No training	Monitoring: offline Feedback: offline	None	**All three groups** improved in: - Trail Making Test A (TMT A); - TMT B; - Memory Diagnostic System (MDS) – Executive Function Quotient (EFQ); Reduction of depression and anxiety symptomatology levels recorded with: - Center for Epidemiologic Study-Depression (CES-D); - State-Trait Anxiety Inventory, State anxiety subscale (STAI-S); Only **SMART group** improved in:- MDS—Working Memory Quotient (WMQ); - MDS—Auditory-verbal working memory (WM) score; Only **Fit Brains**^®^ **group** improved in: - Multifactorial Memory Questionnaire (MMQ)—Contentment (C) subscale.
Pereira-Morales et al. (41)	40 older adults with SMC (**age:** $\bar{x}$ = 66.5 **education:** $\bar{x}$ = 12.3): - 17 in Integrated Psycho-stimulation Program (IPP) group - 12 in Computerized Cognitive Training (CCT) group - 11 in Control group	Randomized controlled trial	**IPP group:** Single-user, asynchronous at-home computerized adaptive multi-domain training with web application (http://app-cerebroactivo.rhcloud.com) AND traditional cognitive exercise through a booklet of cognitive training **Duration:** Thirty-two sessions (60 min, 8 weeks) vs. **CCT group:** Single-user, asynchronous at-home adaptive computerized multi-domain training with web application (http://app-cerebroactivo.rhcloud.com) **Duration:** Thirty-two sessions (60 min, 8 weeks) vs. **Control Group:** Reading of an informative brochure of psychoeducation. **Duration:** 8 weeks	Monitoring: offline Feedback: - web application given verbal-auditory feedback in response to correct and incorrect user answers; - specialist's assistance was gradually reduced across the treatment	None	Only **IPP group** improved in: - Grober and Buschke test (GBT)—Long term memory; - Wechsler Adult Intelligence Scale (WAIS-IV) – Digit and Key Symbols; - Phonological verbal fluency; Reduction of anxiety symptomatology levels and memory complaints recorded with: - STAI—State anxiety subscale; - Subjective Memory Complaints Questionnaire (SMCQ); **Both training groups** (IPP and CCT) improved in: - GBT—Short memory test.
Kumar et al. (42)	82 older adults with SMC (**age:** $\bar{x}$ = 64.0, SD = 4.0)	Pilot study (case series, pre-post design)	Single-user, asynchronous at-home computerized nonadaptive multi-domain training (Virtual Cognitive Health Program provided by MindAgilis) **Duration:** 52 weeks	Monitoring: offline Feedback: offline	None	**At 52 weeks** improvement in: - RBANS – Total index score; - RBANS – immediate memory score; - RBANS – delayed memory score; - RBANS – language score; Reduction of depression and anxiety symptomatology levels recorded with: - Patient Health Questionnaire-9 (PHQ-9); - Generalized Anxiety Disorder (GAD-7); - 86% of the participants reported that the program was at least moderately helpful in improving their cognitive ability.

*$\bar{x}$, Mean; CCT, Computerized Cognitive Training; CES-D, Center for Epidemiologic Studies Depression Scale; GBT, Grober and Buschke Test; IPP, Integrated Psycho-stimulation Program; MDS, Memory Diagnostic System; MMQ, Multifactor Memory Questionnaire; QOL-AD, Quality of Life in Alzheimer's Disease; RBANS, Repeatable Battery for the Assessment of Neuropsychological Status; SD, Standard Deviation; SMART, Smartphone-based brain Anti-aging and memory Reinforcement Training; SMC, Subjective Memory Complaints; STAI, State Trait Anxiety Inventory; TMT, Trail Making Test; WM, Working Memory*.

#### Healthy Older Adults

In recent years, cognitive tele-enhancement interventions in healthy older adults based on remote at-home training assistance have become more popular.

A randomized controlled trial of Brehmer et al. (20) investigated the effects of 5 weeks of intensive domain-general adaptive (e.g., individualized adjustment of task difficulty levels) WM training in comparison to low-level practice (constant low task difficulty level) WM training in younger (age: 20–30 years, education: 15.0 years) and older participants (age: 60–70 years, mean of education: 15.4 years). The tele-enhancement approach consisted of a WM training (Cogmed QM software (https://www.cogmed.com) practiced at-home, administrated for 20–25 sessions over 5 weeks (single-user type and asynchronous). In particular, the training involved the following tasks: (i) maintenance of multiple stimuli at the same time, (ii) short delays during which the representation of stimuli should be held in WM, and (iii) unique sequencing of stimuli order in each trial. Remote offline monitoring was performed and offline feedback on the training performance was provided once a week *via* email for all participants. The benefits of the WM training were evaluated assessing the performance obtained during training and the score achieved in a series of neuropsychological measures. Gains were assessed using criterion, near-transfer, and far-transfer tasks before training, after 5 weeks of intervention, as well as after a 3-month time interval. Group-level analyses showed that younger and older adults improved their performance across the sessions of adaptive WM training. Both younger and older adults gained more in criterion (Digit Span backward and Span Board forward) and near-transfer tasks (Digit Span forward and Span Board backward) after adaptive WM training in comparison to low-level practice WM training. Regarding far-transfer tasks, similar performance improvements for the adaptive and the low-level practice training were observed for tests of interference control (Stroop-color word) and reasoning (Raven's Progressive Matrices). Both younger and older adults gained more after adaptive WM training than after low-level practice WM training in a test measuring sustained attention (PASAT, Paced Auditory Serial Addition Task). Moreover, the individuals that underwent the adaptive WM training reported a higher reduction of memory complaints on the cognitive functioning scale (CFQ). Overall, at post-treatment assessment, younger participants showed greater improvement than older adults in all neuropsychological measures. Interestingly, the observed results were maintained across 3-month follow-up, re-administering the same tests of the baseline.

In a randomized controlled trial, Anguera et al. (43) assessed whether the application of an adaptive custom-designed 3D videogame (Neuroracer), developed to challenge perceptual discrimination and visuomotor tracking, led to enhancements of cognitive control abilities in a sample of healthy older adults (age: 60–85 years, mean of education: 16.8 years). In total, 46 participants were randomized into three groups: Multitasking Training (MTT; *n* = 16), Singletask Training (STT; *n* = 15), and No-Contact Control group (NCC; *n* = 15). For participants in the experimental group (MTT) and in the active control group (STT) a tele-enhancement approach based on Neuroracer was administrated over 12 at-home sessions (over 4 weeks). The modality of the training was single-user, asynchronous, and adaptive (e.g., the difficulty level was adjusted to the individual performance across the training). However, the groups differed for the tasks proposed during the training: the MTT group played only the “Sign and Drive” condition (respond as rapidly as possible to the appearance of a sign only when a green circle was present while maintaining a car in the center of a winding road using a joystick); whereas the STT group performed “Sign Only” condition or “Drive Only” condition. Remote offline monitoring was delivered and participants were contacted weekly by phone or email to encourage and discuss in an offline way their training results. Otherwise, the participants in the NCC group did not receive any treatment. The effects of the training were investigated on training performance measures and on cognitive control domain tasks after 4 weeks from baseline (corresponded to the end of the treatment for MTT and STT groups), and at 6-month follow-up, re-administering the same tests of the baseline. At the group level, the analysis showed that both MTT and STT equally improved their performances across the training sessions. However, at post-training assessment only the MTT group significantly improved in Delayed-recognition working memory and in sustained attention tasks (Test of Variables of Attention, T.O.V.A). Likewise, only the MTT group maintained their improvements on training performance at 6-month follow-up.

In a pilot study (case series pre-post design), Vermeij et al. (40) applied a WM training (Cogmed QM software, https://www.cogmed.com) in 23 healthy older adults (age: 63–81 years, mean of education: 14.4 years) and 18 MCI subjects (age: 59–85 years, mean of education: 13.6 years). Specifically, all participants received 25 sessions (over 5 weeks) of an at-home tele-enhancement approach, administrated in single-user, asynchronous, and adaptive modalities (e.g., the difficulty level was adjusted to the performance of the individual participant by increasing or decreasing the number of items). A control group (active or passive) was not included. Regarding the WM training program, it included a total of 12 exercises (i.e., recall a series of verbally presented numbers and hidden numbers in reverse order; recall a series of verbally presented letters; recall verbally presented letters and their location; recall a series of locations presented, rotating 4 × 4 grid, rotating 3D grid, rotating cube, and rotating circle; recall the order in which moving objects lit up; recall and sort numbers in a 4 × 4 grid; recall the order of presented circles and respond within a certain time frame). In total, eight tasks were chosen for each training session. Remote offline monitoring and weekly offline feedback (given by telephone) were performed. The changes in performance achieved during training as well as on neuropsychological tests (both near-transfer and far-transfer tasks) were considered to quantify the training gains at the end of the treatment and at a 3-month follow-up. During cognitive assessments, parallel versions of tests were administered when available. Furthermore, before the beginning of the treatment, each participant was asked to formulate three personalized goals concerning attention-related or WM-related daily activities. At the end of the treatment and during the follow-up visit each participant was asked to rate his/her personalized goals' performance on a scale from 1 (very far from achieving the goal) to 10 (goal completely achieved), as a subjective measure of training gains. In addition, participants completed the Cognitive Failures Questionnaire in order to evaluate daily life difficulties. Group-level analysis revealed an improvement of training performance for both groups, with larger gains in healthy older adults than MCI. However, both groups showed comparable training gains on near-transfer tasks (WAIS-III Digit Span forward, WAIS-III Digit Span backward, and WMS-III Spatial Span backward) at the post-training assessment. Specifically, healthy older adults showed an improvement on Digit Span forward and on Spatial Span backward tasks, while Digit Span backward and Spatial Span backward scores were enhanced in MCI. Regarding the far-transfer effect, in both healthy older adults and MCI subjects, significant gains on the Ruff Figural Fluency Task (RFFT) were observed. Moreover, both groups reported the achievement of the three personalized goals. The gains on near-transfer tasks and on subjective memory measures were maintained at 3-month follow-up for both groups, whereas only healthy older group maintained the training benefit on RFFT (far-transfer task) at follow-up. Otherwise, at an individual level, a limited number of participants showed reliable training gains only on near-transfer tasks.

In a double-blind randomized controlled trial, Buitenweg et al. (21) investigated whether a 12-week tele-enhancement approach based on a computerized cognitive flexibility training (using games available on website www.braingymmer.com), impacted the cognitive functions in 139 healthy older adults (age: 60–80 years; mean of education: 5.8 years). Participants practiced a single-user type and asynchronous at-home training (60 sessions of 30 min over 12 weeks). Remote offline monitoring was performed, and offline feedback was given weekly or biweekly *via* telephone. Subjects were randomly assigned to three groups: Frequent Switching (FS, *n* = 56), Infrequent Switching (IS, *n* = 33), or Mock Training (MT, *n* = 50). Specifically, for participants in the experimental groups (FS and IS) adaptive games of reasoning, working memory, and attention were selected, but they differed for the frequency of task switching: in each training session, the FS group was asked to perform 10 games of 3 min each, while the IS group was requested to play three games of 10 min each. Otherwise, for subjects in the active control group (MT group), nonadaptive games were chosen, which put minimal demands on executive functions (three games of 10 min each/session). The effects on training performance, as well as on neuropsychological measures, were evaluated after the training and at the 1-month follow-up. In all assessments, parallel tests were used when available. In this study, principal and secondary analyses were conducted, and in both cases the level of significance was adjusted for multiple comparisons (Bonferroni corrections). At the group level, an improvement of training performance across the sessions was found for all groups, although FS and IS groups more significantly improved than the MT group. In addition, all the three groups significantly improved their performances on switching tasks (switch task and dual task), on cognitive flexibility tests (Delis–Kaplan Executive Function System, D-KEFS Trail Making Test-4, TMT-4, and TMT-B- online version), on psychomotor speed measures (Drag-and-drop; Drag-to-grid; Click task; and TMT-A-online version), on processing speed tasks (Digit Symbol Coding, DSC; DSC—online version; Tower of London, ToL), on reasoning measure (Shipley Institute of Living Scale, SILS), on working memory (PASAT), and on verbal long term memory tests (Rey Auditory Verbal Learning Test, RAVLT). The gains in most of the tests that improved post-training were maintained at 1-month follow-up for all groups.

Recently, Rebok et al. (18) in a pilot randomized controlled trial compared the effects of a web-based and classroom-based memory training (10 sessions of 60–75 min of mnemonic skills training over 5–6 weeks). Healthy older adult participants (mean age: 71.1 years; mean of education: 15.5 years) were randomized into three groups: web-based (*n* = 70), classroom (*n* = 69), and wait-list control (*n* = 69) groups. An at-home, single-user type and asynchronous tele-enhancement approach using Active Memory Works (AMW) was administrered to the web-based group. Remote online monitoring was performed and a facilitator was available by phone or Internet for assistance. For the classroom group, online monitoring was applied, and a facilitator was available during in-person mnemonic skills training aimed at remembering lists and sequences of items, text materials, and main ideas and details of stories and other text-based information. The content of the web-based and of the classroom group was matched for the first five sessions focusing on strategy training and for the last five sessions incorporating review and practice of previously learned techniques. Otherwise, the participants in the wait-list control group did not receive any treatment. All three groups completed assessment on objective and subjective measures of cognitive functioning and on everyday functioning scales at baseline, after 6 weeks (corresponded to the end of the treatment for web-based and classroom groups) and at 6-month follow-up. In order to reduce the learning effect, outcome measures included also parallel versions of the Rey Auditory Learning Test (RAVLT) and of the Rivermead Behavioral Memory Test (RBMT). The group-level analysis showed that participants, regardless of group assignment, achieved a significant improvement on objective cognitive tests (RAVLT, RBMT—Paragraph Recall; Fluency test; Animal naming; Word series; and Digit Symbol Substitution test, DSST) and on subjective memory scales (Metamemory in Adulthood, MIA and Memory Functioning Questionnaire, MFQ). The gains were maintained for all three groups at the 6-month follow-up. Moreover, the web-based and classroom-based training groups reported comparable satisfaction with the program. In conclusion, this study suggested that web-based training is an acceptable and feasible mode to provide memory training in healthy older adults and its effects are comparable to in-person training.

#### Subjective Memory Complaint

Investigation of the efficacy of cognitive training delivered remotely has also been extended to subjects who experience a subjective decline in cognitive functions.

In this regard, Oh et al. (38) in a randomized controlled trial study, examined the effects of 8 weeks of a tele-enhancement approach based on smartphone cognitive training in participants that reported memory difficulties at Subjective Memory Complaints Questionnaire (SMCQ). A total of 53 SMC adults (age: 50–68 years; education: 6–18 years) were randomly assigned to one of three groups: Smartphone-based brain Anti-aging and Memory Reinforcement Training group (SMART, *n* = 18), Fit Brains® group (*n* = 19), and wait-list control group (*n* = 16). For SMART and Fit Brains® groups the intervention was administrated on smartphone for 40 sessions over 8 weeks and was in single-user and in asynchronous modality. Remote offline monitoring was performed and offline feedback was provided weekly *via* phone calls. Specifically, the SMART training included two nonadaptive and eight adaptive tasks aimed to improve attention, memory, and working memory abilities; whereas the Fit Brains® treatment (www.fitbrains.com) included adaptive tasks for several cognitive domains (e.g., attention, memory, and logic). Otherwise, no cognitive intervention was provided to participants in the wait-list control group. Training gains were evaluated on neuropsychological tests and on self-reported questionnaires on subjective memory complaints, depression, and anxiety. Group-level analyses showed that all participants, regardless of group assignment, improved in terms of the Trail Making Test (part A and B) and the MDS—Executive Functions Quotient (EFQ). Moreover, we found a decrease in depression and anxiety levels, evaluated with the Center for Epidemiologic Study-Depression (CES-D), and State-Trait Anxiety Inventory, State anxiety subscale (STAI-S) scales, respectively. Only the SMART group significantly improved their performance on WM tests (Memory Diagnostic System, MDS—Working Memory Quotient, WMQ and MDS—Auditory-verbal working memory, WM score). Otherwise, a significant improvement on a self-report questionnaire [Multifactorial Memory Questionnaire, MMQ—Contentment (C) subscale] was found only in the Fit Brains® group.

In a randomized controlled trial study, Pereira-Morales et al. (41) investigated the effectiveness of a computerized cognitive training program in a group of 40 older adults with subjective memory complaints, evidenced at the SMCQ (mean age: 66.5 years; mean of education: 12.3 years). In this study, participants were randomly assigned to an Integrated Psychostimulation Program (IPP, *n* = 17), to a Computerized Cognitive Training (CCT, *n* = 12), or to a control group (*n* = 11). All groups performed their activities over 8 weeks in at-home, single-user, and asynchronous modalities. Only for experimental groups (IPP and CCT) the computerized training was adaptive (e.g., adjustment of difficulty level on user's was performances) and remote offline monitoring was performed. Verbal-auditory feedback was given by web application and specialist's assistance was gradually reduced across the treatment sessions. The IPP group underwent a tele-enhancement approach based on computerized cognitive training associated with traditional pen and paper cognitive training. The IPP training session comprised 60 min of a multi-domain (e.g., orientation, attention, memory, and executive functions) computerized cognitive training through a web application (http://app-cerebroactivo.rhcloud.com) associated with 30 min of traditional pen and paper exercises with a booklet of cognitive training (BCT). Otherwise, the CCT group received 60 min of a tele-enhancement approach based only on computerized cognitive training, using the same web application used for the IPP group. Finally, the control group was asked to read a psychoeducational informative brochure. Following the intervention, psychological, and neuropsychological measures, were administered and analyzed using group-level analyses and multiple testing correction (Bonferroni). The results indicated that the IPP group significantly improved on long-term memory (Grober and Buschke test, GBT), processing speed (Wechsler Adult Intelligence Scale, WAIS-IV—Digit and Key Symbols), and phonological verbal fluency measures. Moreover, this group showed a reduction of anxiety symptomatology level (STAI) and of memory complaints (SMCQ). Both IPP and CCT groups showed a significant improvement on a short-memory test (GBT). Otherwise, for the control group, no statistical changes were found on cognitive and clinical measures. In conclusion, these findings demonstrated that intensive training that combined computerized cognitive training and traditional cognitive training led to significant improvements in cognitive function and psychological well-being.

Kumar et al. (42), in a pilot study (case series pre-post design), evaluated in a group of 82 older adults with subjective cognitive decline (SMC, age: 60.0–74.9) the impact of an intensive (52 weeks) multidomain lifestyle intervention (Virtual Cognitive Health, VC Health program) on cognitive functions and mental health. Participants were enrolled in the study if reported subjective cognitive decline as recorded on the Subjective Cognitive Decline Questionnaire, SCD-9. A control group (active or passive) was not included. In particular, the VC Health program consisted of a tele-enhancement approach based on computerized training focused on nutrition, physical exercise, and cognitive training, designed to prevent or delay cognitive decline and impairment in older at-risk adults. More specifically, the computerized cognitive training program (provided by MindAgilis; London, England) was nonadaptive and was focused on processing speed, executive functions, working memory, episodic memory, and mental speed. Moreover, it was administrated at-home and was single-user type and asynchronous. Furthermore, remote offline monitoring and offline feedback (*via* telephone, email, and/or text messaging) were performed. The effects were evaluated during the treatment (at 24 weeks) and post-training (after 52 weeks) in terms of cognitive and mental health measures, using alternate forms of the tests when available (i.e., Repeatable Battery for the Assessment of Neuropsychological Status, RBANS). Group-level analysis showed a decrease in depression symptoms level (as measured by Patient Health Questionnaire, PHQ-9) at 24 weeks. At the end of the treatment (52 weeks) a significant improvement on RBANS-Total Index score and on immediate memory, language, and delayed memory RBANS sub-Index scores were observed. Furthermore, a decrease in depression (PHQ-9) and anxiety levels (Generalized Anxiety Disorder, GAD-7) was also recorded. Lastly, the 86% (51/59) of the participants who completed the treatment (*n* = 59) reported that the VC Health program was at least moderately helpful in improving their cognitive ability.

## Discussion

To our knowledge, there are currently no other reviews that have investigated the use of cognitive enhancement treatments carried out with remote control on cognitively healthy older adults or on SMC subjects. The main purpose of the present review was to highlight studies that have used tele-enhancement and where the therapist supplied feedback on training performance to provide a general overview of treatments conducted at patient homes with remote control in healthy older adults and SMC subjects.

Only eight studies that adopted at-home tele-enhancement cognitive interventions were included in this review; specifically, five focused on cognitively healthy older adults, while three focused on participants with SMC. More specifically, only studies with double-loop communication between the clinic and home were considered because the presence of feedback on cognitive performance seems to have many benefits, especially in terms of participant motivation (41).

The results were heterogeneous due to some substantial differences between the studies. The cognitive enhancement protocols adopted by the studies included in this review focused primarily on the enhancement of different cognitive domains. In particular, some studies applied an intervention directed to specific cognitive function (18, 20, 40, 43) while the others reported the effects of a multi-domain intervention aimed at enhancing working memory, attention, reasoning, executive functions, episodic memory, and orientation (21, 38, 41, 42).

Regarding the studies on healthy older adults, three studies investigated the effects of a tele-enhancement intervention using memory training (18, 20, 40): one study applied a computerized cognitive training directed to cognitive control abilities (43), while another one applied a multi-domain cognitive training (21). Despite the differences in methodologies, types of intervention, and sample sizes, all investigations on cognitively healthy older adults found evidence for an immediate improvement on objective and/or subjective cognitive outcome measures and reported long-term effects during follow-up evaluations, demonstrating general maintenance of improvements (from 1 to 6 months).

Regarding the studies on participants with SMC, all three studies focused on the application of multi-domain tele-enhancement interventions and reported improvements in different cognitive domains, such as memory, executive functions, processing speed, and language (38, 41, 42). Moreover, a reduction in anxiety and depression symptomatology levels, as well as in subjective memory difficulties, was described in all studies (38, 41, 42). Unfortunately, none of the studies involving participants with subjective memory complaints assessed the maintenance of cognitive tele-enhancement effects over time.

However, a strength of most of the included studies was the implementation of adaptive training, which allows for reaching a balance between individual abilities and the difficulty level of rehabilitative activities, implementing a personalized treatment (20, 21, 38, 40, 41, 43). Although these results are of considerable interest, it is important to note some study limitations.

The existing research reflects a general lack of randomized clinical trials, a small sample size, a great diversity of technology utilized, and lack of common outcome measures. Moreover, selected studies were widely different in study design and cognitive treatments (number, duration and frequency of sessions, mode of tele-enhancement intervention delivery, monitoring, and feedback modality). In addition, only a few studies applied a multiple comparisons correction in the statistical analyses (21, 41). Taken together, these differences make it difficult to compare the results of the studies and to draw a final conclusion. In addition, the characterization of the cognitive profiles of the enrolled subjects was not always detailed. Since the literature has shown that subjective cognitive decline and mild cognitive impairment are associated with an increased risk of developing dementia (44–47), it is necessary to investigate in depth the cognitive profile and the presence of subjective cognitive complaints in the recruited subjects. This evidence underlines the need for future well-designed studies. Despite these critical issues, telerehabilitation appears to be an excellent opportunity for intervention to strengthen cognitive functions, with results comparable to face-to-face rehabilitation interventions. Telerehabilitation presents new opportunities to enhance cognition in older adults and in subjects with subjective memory complaints. Future studies will involve a large cohort of subjects and will allow us to evaluate whether tele-enhancement approaches could be as effective as face-to-face intervention.

## Author Contributions

CA, EC, MS, EG, FB, FR, GB, OZ, RM, and MC: conception and methodology and writing—review and editing. CA, EC, RM, and MC: data curation. CA, EC, MS, EG, RM, and MC: writing—original draft preparation. All authors have read and agreed to the published version of the manuscript.

## Conflict of Interest

The authors declare that the research was conducted in the absence of any commercial or financial relationships that could be construed as a potential conflict of interest.
